# Data analysis plan of the OECD PaRIS survey: leveraging a multi-level approach to analyse data collected from people living with chronic conditions and their primary care practices in 20 countries

**DOI:** 10.1186/s13104-024-06815-7

**Published:** 2024-06-06

**Authors:** Peter Groenewegen, Peter Spreeuwenberg, Rob Timans, Oliver Groene, Rosa Suñol, Jose Maria Valderas, Mieke Rijken

**Affiliations:** 1 Nivel, Netherlands Institute for Health Services Research, PO Box 1568, Utrecht, 3500BN Netherlands; 2 Vice Chair of the Board, OptiMedis AG, Hamburg, Germany; 3 Department of Management and Entrepreneurship, Faculty of Business, Economics and Society, University of Witten/Herdecke, Witten, Germany; 4 Avedis Donabedian Research Institute, Barcelona, Spain; 5 Red de investigación en servicios de salud en enfermedades crónicas (REDISSEC), Barcelona, Spain; 6 Health Services & Policy Research Group, University of Exeter, Exeter, UK; 7 Centre for Research in Health Systems Performance, Yon Loo Lin School of Medicine, Department of Family Medicine, National University of Singapore, National University Health System, Singapore, Singapore; 8 Department of Family Medicine, National University Health System, Singapore, Singapore; 9 Department of Health and Social Management, University of Eastern Finland, Kuopio, Finland

**Keywords:** International comparison, Multilevel analysis, Primary care, Health services research

## Abstract

**Objective:**

In view of the increasing number of people with (multiple) chronic conditions, the Organisation for Economic Co-operation and Development (OECD) initiated the International Survey of People Living with Chronic Conditions (PaRIS survey), which aims to provide insight in patient-reported outcomes and experiences of chronic care provided by primary care practices to support policy development. The objective of this research note is to describe the structure of the data, collected in the PaRIS survey and how the data will be analysed in a multilevel approach for cross-country comparison.

**Analysis plan:**

The data structure of the PaRIS survey represents three levels: countries/health systems, primary care practices and patients. Multilevel analysis is used because of its accuracy in estimating country-level outcomes, its flexibility in modelling relationships, and its opportunities in connecting to relevant policy questions. Country-level outcomes will be estimated to facilitate cross-country comparison and (future) within-country comparison over time. Characteristics of patients that potentially explain variation in patient-reported outcomes and experiences can be linked to primary care practice and country/health system characteristics. This makes it possible to address policy-relevant questions relating, e.g., to the impact of chronic care management on patients with a specific chronic condition.

## Background

The number of people with chronic conditions is increasing. This requires adaptations of health systems, in particular towards increasing their people-centredness, integrated care and a stronger role of primary care. An international evidence base for developing policies regarding health system adaptations is lacking. How health systems currently perform from the perspective of people living with chronic conditions has not been studied systematically for a larger number of countries. To this end, the Organisation for Economic Co-operation and Development (OECD) initiated the International Survey of People Living with Chronic Conditions (PaRIS survey), aiming to provide insight in patient-reported outcomes and experiences of chronic care provided by primary care (PC) practices [1]. The PaRIS-SUR consortium has been tasked by the OECD to support the development and implementation of the survey. The PaRIS survey has been developed inclusively with stakeholders to ensure relevance and uptake of its results [2]. 

The PaRIS survey consists of a patient questionnaire for PC service users aged 45 and over about their care experiences, perceived outcomes and socio-demographic characteristics (https://www.oecd.org/health/paris/PaRIS-patient-questionnaire.pdf), and a practice questionnaire for PC practices (PCPs) about the characteristics and processes of chronic care management (https://www.oecd.org/health/paris/PaRIS-patient-questionnaire.pdf). To facilitate international comparisons and cross-country learning, the PaRIS survey uses validated standardised instruments and procedures for sampling, data collection and analysis. The PaRIS survey research questions (RQs) relate to (RQ1) cross-country comparisons of patient-reported outcomes and (RQ2) experiences of care, and how the patient-reported outcomes and experiences relate to (RQ3) patient characteristics such as their age, gender, health-risk behaviours, and confidence in self-managing their health and care; (RQ4) characteristics and processes of chronic care management within PCPs; and (RQ5) key characteristics of health systems and countries.

The first data collection cycle of the PaRIS survey took place in 20 countries in 2023 and early 2024. In consultation with its Member States, the OECD is planning further data collection in the future, as well as extension to other countries.

The main reason for submitting this analysis plan for publication was – comparable to the publication of study protocols – that it increases the transparency of planned analyses. As far as we know, there are only few international comparative studies with patient-reported outcome and experience measures (PREMs and PROMs). Given the hierarchical structure of the data, with patients nested in PCPs, nested in countries, we propose the use of state-of-the-art methodology, namely multilevel analysis. By publishing this analysis plan, we open up for criticism, provide relevant information for researchers who want to use the data for secondary analysis and a helpful example to other researchers.

## Data analysis plan: a multilevel data structure of patients, PCPs and countries

The core of the data analysis plan of the PaRIS survey, and the focus of this research note, is the multilevel structure of the data and the use of multilevel analysis (MLA) to analyse the data according to state of the art methodology [3]. The actual analyses will be done in Stata and in MLwiN. The use of MLA facilitates the link to policy at country level and action at practice level in order to improve patients’ experiences and outcomes. In this research note we describe the structure of the data and how we will analyse them in a multilevel approach. Other important elements of the data analysis plan, such as data cleaning, handling of missing data and scale construction, will be described in a technical report.

### Data structure

**Fig. 1:**
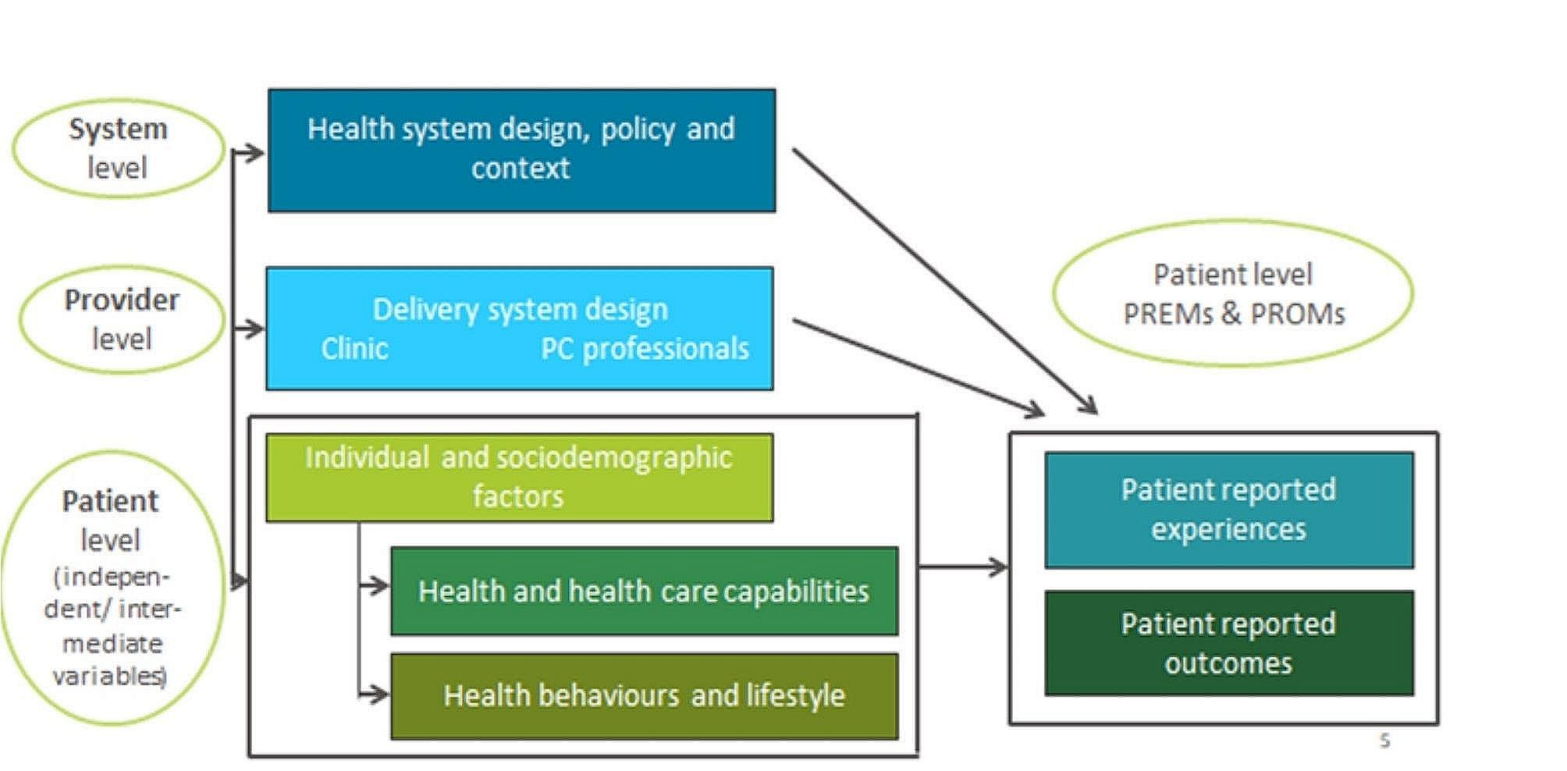
Elements of the PaRIS-SUR conceptual model and three levels of data collection and analysis

The data structure of the PaRIS survey represents three levels: countries/health systems, PCPs and patients. As it is designed for comparison between countries/health systems, they form the highest level (level 3). Characteristics of the PC systems and the broader (healthcare) context are available from OECD sources, including the Health Systems Characteristics Survey (Health Systems Characteristics Survey (oecd.org). The survey among PCPs (level 2) collects data on the practice characteristics and the care provided, in particular related to chronic care management [4]. The main instrument is a survey among PC service users aged 45 years or older to collect patient-reported data (level 1) [5]. The sampling design is structured accordingly. Service users are sampled from the patients of the participating PCPs. The PaRIS survey thus has a nested design (see Fig. 1): PC service users (target response 75 patients per PCP) are nested in PCPs (target response 100 PCPs per country), which are at their turn nested in countries/health systems (20 countries in the current PaRIS survey) [6]. 

### Variation between countries

Country level values of the PREMs and PROMs (dependent variables) will be estimated in a MLA, to take the clustering of patients within practices within countries into account (RQ1 and RQ2). Policy-makers will need information first of all about the position of their country relative to the other countries. For a fair comparison, the population of all countries will be made comparable through a uniform standard population across all participating countries. With an eye to future measurements of PROMs and PREMs they will also need the information for their own country’s specific population. Therefore, estimates will be generated for country-specific reference populations and for a uniform standard population to facilitate country-specific as well as cross-country learning.

For each dependent variable, a multilevel regression model will be specified (see Eq. (1) below). The country values will be estimated based on the overall average (β_0_) + the country level residual (v_0k_). Standardisation variables and additional variables, such as administration mode or language of the questionnaire (both of which vary in some countries), and (if necessary) case-mix adjusters [7] will be added as *n* independent variables (marked blue in (1)).

y_ijk_ = β_0_ + β_(n)_ x_(n)ijk_ + v_0k_ + u_0jk_ + e_0ijk_(1)

Here *i* indicates the individual patients, *j* the PCPs, and *k* the countries. The residuals at PCP and patient level are u_0jk_ and e_0ijk_ respectively. The target for responding PCPs and patients per country is large enough to assess the main outcome measures reliably at the country level and to compare groups of countries.

The reference population of PCPs and eligible patients will be defined for each country, based on information provided by the National Project Managers (NPMs) in their sampling report. Additionally, a reference population of PCPs and eligible patients will be defined across all participating countries in consultation with the OECD.

The analysis to arrive at cross-country comparable scores is a MLA using rescaled standardisation variables. The standard population could, e.g., be defined as a population of patients consisting of 45% men and 55% women, with 30% aged 45 to 54, 30% 55 to 64, 25% aged 65 to 74, and 15% aged 75 or over, of whom 30% receive PC from a solo practice and 70% from a group practice.

The scores on outcome variables for all countries are estimated from the regression model in Eq. (1). Without adjustment for the composition of the population, the overall average β_0_ would be estimated with all standardisation variables having the value 0. Changing the coding of the standardisation variables to represent the standard population will thus influence the value β_0_. To illustrate this, assume there is one standardisation variable ‘patients’ gender’ (Pgen) included, with two categories: 0 for women and 1 for men. With this coding, β_0_ is the average based on Pgen = 0, which is the average for women. So, the effect of men is partialled out (the average for men is the sum of β_0_ and the effect of Pgen, β_(n)_). By rescaling the values of Pgen, the interpretation of β_0_ changes and represents the defined standard population [8]. With 55% of the intended standard population being women and 45% being male, we rescale the original 0–1 coding of Pgen for women (originally coded as 0) into 0-0.55= -0.45; and for men into 1-0.45 = 0.55. The result is that β_0_ now reflects the weighted overall average, i.e. weighted for the distribution of patients’ gender in the standard population. In this way, relevant characteristics for weighting of country populations can be incorporated in the MLA to impact the overall average and the country scores on the selected outcome variables.

### Variation by socio-demographic characteristics

To assess the variation in PROMs and PREMs by a number of background characteristics (RQ3), we will estimate multilevel regression models with random intercepts at practice and country level and fixed effects at patient level (marked blue in Eq. (2) below). The number of participating patients is large enough to estimate the effects of multiple patient-level independent variables simultaneously (*m* in Eq. (2)). Using a standard population (as with RQ1 and RQ2) is not necessary, because the focus is on the coefficients of the socio-demographic characteristics and the standardisation variables will be included in these.

Y_*ijk*_ *=* β_*0*_ *+* β_*(m)*_*x*_*(m)ijk*_*+ v*_*0k*_*+ u*_*0jk*_*+ e*_*0ijk*_(2)

The selection of patient characteristics will be guided by theoretical notions, a scoping review of relevant literature, and the information participating countries and the OECD need for policy development. The inclusion of the patient-level variables may not only impact the patient-level variance component, but also the variance at practice and country level. Changes in variation at the higher levels indicate differences in composition of the responding patient group between practices and countries.

### Variation by PCP characteristics

The analysis of PCP characteristics RQ4) will build on the analyses described above by including a set of patient characteristics at the individual level that have a significant relationship with the PREMs and PROMs. We will estimate multilevel models with random intercepts at practice and country level (*v*_*0k*_ and *u*_*0jk*_ ) and fixed effects at practice level (marked red) and patient level (marked blue; see Eq. (3) below).

y_*ijk*_ *=* β_*0*_ *+* β_*(m)*_*x*_*(m)ijk*_ *+* β_*(p)*_*x*_*(p)jk*_*+ v*_*0k*_*+ u*_*0jk*_*+ e*_*0ijk*_(3)

The selection of practice level variables will be guided by theoretical notions and empirical evidence as well as the main policy interests and information needs of participating countries and the OECD. Depending on theoretical considerations and these interests and information needs, we will also estimate some cross-level interactions, particularly suited to explore policy relevant constellations of health system or PCP characteristics and patient characteristics. For example, if health authorities in the participating countries would be interested to see whether certain elements of chronic care management (PCP level) show different results for, e.g., older and younger patients or patients with certain chronic conditions (patient level), a cross-level interaction is indicated. Another example is that some of the patient level socio-demographic variables relate to health behaviours and self-management capabilities. As these characteristics are amenable to (policy) interventions at country or PCP level, again cross-level interactions are indicated.

### Variation by health system/country characteristics

Finally, we will estimate multilevel models with random intercepts at practice and country level, fixed effects at patient level and practice level (marked blue), and a fixed effect at country level (marked red) in Eq. (4) (RQ5).

y_*ijk*_ *=* β_*0*_ *+* β_*(m)*_*x*_*(m)ijk*_ *+* β_*(p)*_*x*_*(p)jk*_ *+* β_*(q)*_*x*_*(q)k*_*+ v*_*0k*_*+ u*_*0jk*_*+ e*_*0ijk*_ (4)

Although a substantial number of countries are participating (*N* = 20), their number is still small (see for an extended discussion of the number of countries needed Bryan and Jenkins [9]. The power of the analysis at country/system level is not determined by the number of patients or PCPs but by the number of countries. This allows for inclusion of only one country/system level characteristic at a time. It is not possible to estimate the effects of multiple characteristics of the health system and/or confounders (such as the wealth of countries) simultaneously on the PREMs and PROMs. Considering that more countries are expected to participate in subsequent cycles, more opportunities for analysis of health system/country characteristics will arise. As with the variation by PCP characteristics, it is possible to analyse policy relevant cross-level interactions.

## Limitations

We will discuss three limitations of the design of the PaRIS survey.

The first limitation relates to *sampling*. Firstly, the statistical analysis assumes random samples at all levels. In the PaRIS survey, random samples of PCPs are drawn and within each PCP a random sample of patients is drawn. However, the participating countries are not a random sample. This is not unique for the PaRIS survey [10], but it implies that the findings cannot be generalised to a defined population of countries, such as all OECD countries.

The second limitation refers to the use of *reference populations*. This requires information that is most probably not available in the same detail for all countries. The target population of patients consist of people of 45 years or older, who have had contact with a PCP during the half year preceding sampling. This makes it more complicated to define a reference population than for a survey that samples from the general population. Also, the characteristics of the population of PCPs are not available in all countries. For cross-country comparison, the reference population could consist of the average population and PCPs of the participating countries or data, provided by the NPMs. For the within-country comparison over time, more detailed information will be available. Using the two reference populations, we provide as much relevant information as possible for countries to develop evidence-informed policies. The process is transparent and will be detailed in a technical report that contains the respective reference populations.

Finally, we will analyse dependent variable scales that will be constructed based on guidelines developed for analysis in single level models. This is not optimal, given the structure of the data. The alternative is a *latent variable MLA*, where the latent variable scale value for an individual is model-based, using the combination of the valid individual scores on the items and the relation between all items and the latent variable over all cases. This takes the multi-level structure of the data into account, and as such generates more precise scores for individual patients, nested in PCPs and countries [3, 11]. We have chosen for scale construction in single level models to be able to apply the standard rules (e.g. regarding missing values and cut-off points) for validated scales included in the patient questionnaire (in particular, PROMs and PREMs that are frequently being used in international studies) to assure comparability with published results from these validated scales. Further research is needed to incorporate established guidelines from validated scales into multilevel latent variable analysis [3, 12]. 

Apart from these limitations, the strength of the PaRIS survey lies in the possibility to link patients, PCPs and countries and to use MLA. We are able to determine the variation on patient, PCP and country/health system level, and to identify factors on these levels that may affect outcomes and experiences. The sampling of actual users of PC services instead of the general population also guarantees that we are asking people who have actual experiences.

## Data Availability

Not applicable; data are currently being collected.

## Abbreviations

OECD: Organisation for Economic Co-operation and Development
PaRIS survey: International Survey of People Living with Chronic Conditions
MLA: Multilevel analysis
NPMs: National Project Managers
PREMs: Patient-reported experience measures
PROMs: Patient-reported outcome measures
Pgen: Patients’ gender
PC: Primary care
PCPs: Primary care practices
RQs: Research questions
